# An Open Receptor-Binding Cavity of Hemagglutinin-Esterase-Fusion Glycoprotein from Newly-Identified Influenza D Virus: Basis for Its Broad Cell Tropism

**DOI:** 10.1371/journal.ppat.1005411

**Published:** 2016-01-27

**Authors:** Hao Song, Jianxun Qi, Zahra Khedri, Sandra Diaz, Hai Yu, Xi Chen, Ajit Varki, Yi Shi, George F. Gao

**Affiliations:** 1 CAS Key Laboratory of Pathogenic Microbiology and Immunology, Institute of Microbiology, Chinese Academy of Sciences, Beijing, China; 2 University of Chinese Academy of Sciences, Beijing, China; 3 University of California, San Diego, La Jolla, California, United States of America; 4 University of California, Davis, Davis, California, United States of America; 5 Research Network of Immunity and Health (RNIH), Beijing Institutes of Life Science, Chinese Academy of Sciences, Beijing, China; Institut Pasteur, FRANCE

## Abstract

Influenza viruses cause seasonal flu each year and pandemics or epidemic sporadically, posing a major threat to public health. Recently, a new influenza D virus (IDV) was isolated from pigs and cattle. Here, we reveal that the IDV utilizes 9-O-acetylated sialic acids as its receptor for virus entry. Then, we determined the crystal structures of hemagglutinin-esterase-fusion glycoprotein (HEF) of IDV both in its free form and in complex with the receptor and enzymatic substrate analogs. The IDV HEF shows an extremely similar structural fold as the human-infecting influenza C virus (ICV) HEF. However, IDV HEF has an open receptor-binding cavity to accommodate diverse extended glycan moieties. This structural difference provides an explanation for the phenomenon that the IDV has a broad cell tropism. As IDV HEF is structurally and functionally similar to ICV HEF, our findings highlight the potential threat of the virus to public health.

## Author Summary

Of the *Orthomyxoviridae* family of viruses, influenza A, B and C viruses all can cause disease in humans. Recently, a novel influenza D virus (IDV) with approximately 50% amino acid identity to human influenza C virus (ICV) is found in pigs and cattle. This novel virus can establish infection in other mammals including ferrets and guinea pigs. However, the cellular receptor for viral entry and the molecular mechanism for its broad host range is unclear. We performed combined structural and functional studies on the viral surface protein, hemagglutinin-esterase-fusion (HEF), and demonstrated that IDV (like ICV) uses 9-O-acetylated sialic acid as its receptor, but the IDV HEF has an open receptor-binding cavity to accommodate diverse extended glycan moieties. Our findings reveal in exquisite detail how the receptors or substrates bind to the receptor-binding site or esterase active site, providing a clue for the development of novel therapeutics against the conserved esterase pocket. Furthermore, the IDV HEF can bind human trachea epithelia, indicating that the IDV virus may become a potential threat to public health.

## Introduction

Influenza viruses are enveloped, segmented, single-stranded, negative-sense RNA viruses and belong to the family *Orthomyxoviridae* [1]. The genomes of influenza A virus (IAV) and influenza B virus (IBV) consist of eight RNA segments, whereas influenza C viruses (ICV) only have seven segments. Both IAV and IBV contain two major surface glycoproteins: the hemagglutinin (HA), which binds to sialylated host cell receptors and mediates membrane fusion; and the neuraminidase (NA), which destroys the receptor by cleaving sialic acid from host cell membranes, thereby releasing newly assembled virus particles [1], and likely assisting initial invasion by destroying sialylated mucin decoys [2]. ICV, however, has only one major surface glycoprotein, the hemagglutinin-esterase-fusion (HEF) protein, which possesses all-in-one of receptor binding, receptor destroying and membrane fusion activities [3, 4]. While IAV infects avian, human, swine, and many other mammalian species including dogs, horses, tigers and seals, IBV and ICV are found principally in humans and rarely infect other species [3].

ICV usually causes mild upper respiratory tract infections in children with cough, rhinitis and rhinorrhea as clinical symptoms [5, 6]. The virus only occasionally spreads to the lower respiratory tract and causes bronchitis, bronchiectasie and broncho-pneumonia [7]. Encephalopathy has also been occasionally reported [8]. Seroepidemiological studies have revealed that ICV is widely distributed globally and that the majority of humans acquire antibodies against the virus early in life [9, 10]. Aside from humans, there is evidence that ICV possesses the ability to infect animals [3]. Serological studies showed that antibodies against ICV are present in pigs [11–13]. In 1981, fifteen strains of ICV were isolated from domestic pigs in China [14], which showed characters highly related to viruses isolated from humans in Japan [15, 16]. Furthermore, pigs have been shown to be susceptible to experimental infection with both pig and human ICVs, and the virus is able to be transmitted from the infected to uninfected contact pigs [14], suggesting that interspecies transmission of ICV between humans and pigs might occur in nature. Dogs may also serve as a natural reservoir for human ICV due to the presence of viral replication and clinical symptoms in experimental infections and the prevalence of antibody to ICV among dogs [12, 17–19].

In 2011, an influenza C-like virus was isolated from swine in Oklahoma (D/swine/Oklahoma/1334/ 2011 [D/OK]) exhibiting influenza-like symptoms [20]. The genome of this virus also contains seven segments, and sequence analysis showed approximately 50% overall amino acid homology to either human or previous swine ICVs. D/OK did not cross-react with antibodies against human ICVs and, importantly, was unable to reassort with human ICVs or generate viable progeny [20–22]. However, the low seroprevalence rate observed in both swine and humans to D/OK (9.5% and 1.3%, respectively) suggested that swine and humans are not likely to be a major reservoir of this novel virus [20]. Subsequent serological studies have showed that antibodies against D/OK are almost ubiquitously present in cattle, and several novel D/OK-like virus strains have been isolated from cattle with respiratory disease which could be divided into two distinct lineages represented by D/OK and D/bovine/Oklahoma/660/2013 (D/660) [21, 23]. These two genetic and antigenic distinct clades have been shown to reassort with each other [23]. In addition, D/OK has a broader cell tropism than human ICV and is capable of infecting ferrets, pigs and guinea pigs and transmit to naive animals by direct contact [20, 24]. Based on these differences to ICV it was suggested that this virus warrants classification as a new genus of influenza virus, named influenza D virus (IDV) with cattle as the potential reservoir [21]. Subsequently, more IDVs or viral genomic segments were identified from China and France in cattle, suggesting the wide geographic distribution of IDV [25, 26]. Interestingly, IDV is common in clinical samples of bovine respiratory disease complex (BRDC), which is the leading cause of morbidity and mortality in feedlot cattle [23, 27]. BRDC is a challenging multi-factorial disease caused by viral, bacterial pathogens and environmental factors, leading to severe clinical signs and deaths [28]. IDV was detected in clinical BRDC samples, co-infected with bovine coronavirus (BCV), bovine viral diarrhea virus (BVDV), bovine respiratory syncytial virus (BRSV), bovine herpesvirus 1 (BHV-1), and *Pasteurella multocida*, *Mannheimia haemolytica*, *Histophilus somni et al*, suggesting IDV has the pathogenic potential in BRDC [23]. In addition, a latest serological study showed that antibodies to IDV were present in sheep and goats in United States, suggesting that small ruminants are also susceptible to IDV infection [29].

To further evaluate the infectivity and transmissibility of IDV, we expressed and purified the ectodomain of D/OK HEF, and determined that it also uses 9-O-Acetyl-Sia as its receptor by glycan microarray. We also solved the crystal structure of D/OK HEF, both in its native state (resolution of 2.4 Å) and in complex with its receptor analogue (resolution of 3.1 Å), and the structure of the enzymatically inactive HEF (resolution of 2.4 Å) alone and in complex with two receptor analogs respectively (both resolutions of 2.2 Å). Indeed, our results show that IDV HEF is functionally and structurally similar to ICV HEF, but with some distinct characteristics.

## Results

### IDV HEF uses 9-O-Acetylated-Sia as its receptor

**Fig 1:**
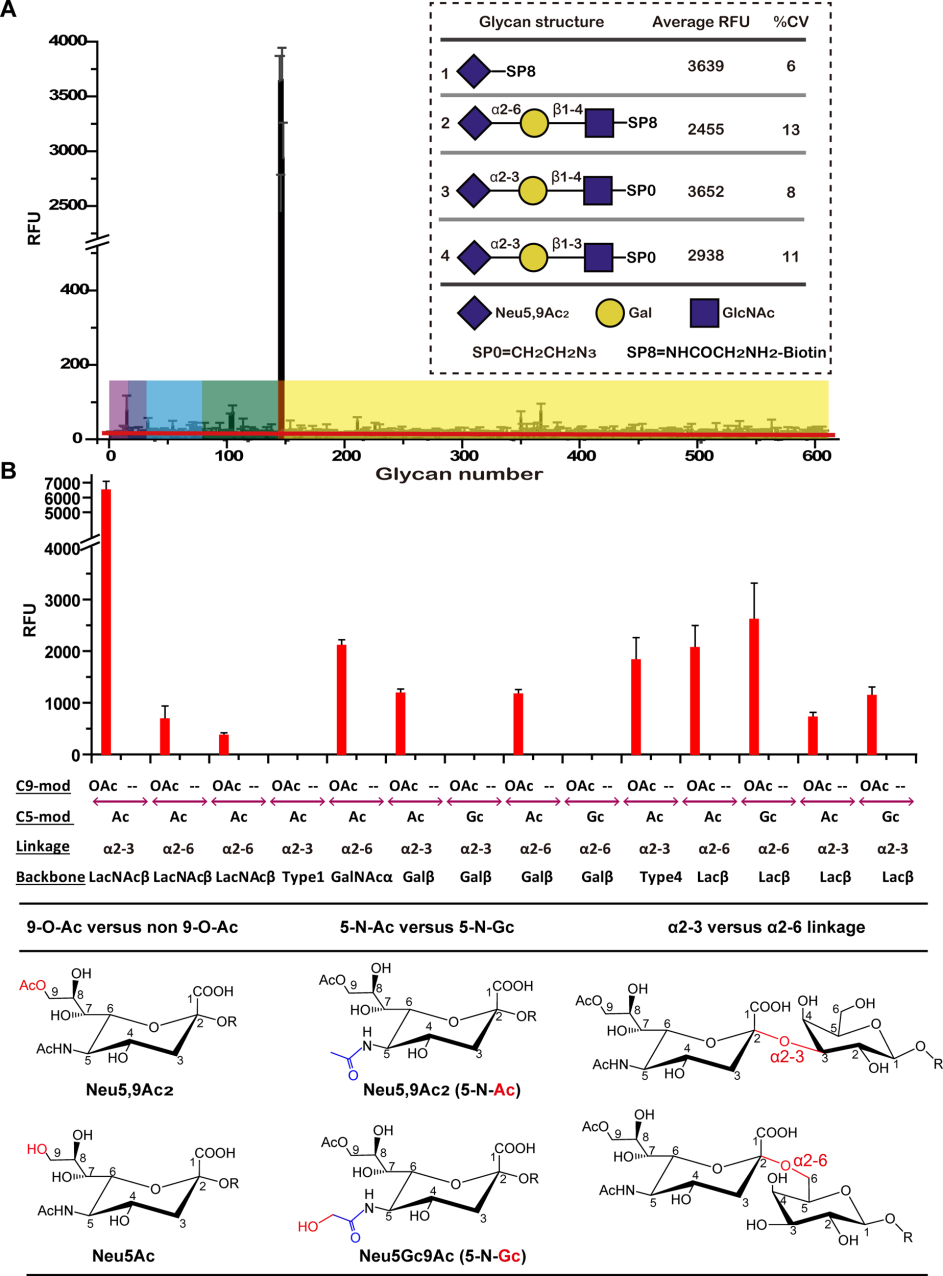
Receptor specificity of the IDV HEF protein. (A) Glycan microarray analyses of the IDV enzymatically inactive HEF (HEF-mut) protein using the CFG glycan microarray. Binding to different types of glycans on the array is highlighted, where magenta represents Neu5Gc, blue represents α2-8-ligands, cyan represents α2-6-ligands, green represents α2-3-ligands, red represents Neu5,9Ac_2_ and yellow represents other glycans. The HEF-mut protein displayed a good avidity to Neu5,9Ac_2_ ligands. The structure formulas of the top representative are shown. Error bars represent the standard deviation (SD) of the mean value. Percent coefficient of variation (%CV) = 100×standard deviation/mean. A %CV of less than 50% indicates data reliability. (B) Glycan microarray analysis of IDV HEF-mut proteins using a more extended paired array. The library of glycans tested was designed to test the influence on protein binding of (1) glycosidic linkage of 9-O-Ac-Sia (α2–3 versus α2–6) and (2) modifications at C5 (N-acetylation, 5-N-Ac, versus N-glycosylation, 5-N-Gc). Data presented as mean ± SD.

The ectodomain of HEF from D/OK strain was cloned and expressed using a baculovirus expression system based on a previously reported method [30–33] with slight modifications. To avoid the enzymatic cleavage of the receptor substrates, we generated the catalytic mutant for the binding experiments and for the structures of the relevant complexes. Previous studies have demonstrated that the residues S57, D356 and H359 create a catalytic triad in ICV HEF esterase, which has been proved by site-directed mutagenesis and structural analysis [34, 35], and the sequence alignment between the ICV and IDV HEF proteins reveals that a highly conserved catalytic triad is also observed in IDV HEF protein (S2 Fig). Thus, we designed the enzymatically inactive HEF protein (HEF-mut) containing S57A, D356A and H359A substitutions, and expressed it using the same method as wild type protein. Soluble protein was purified by metal affinity chromatography followed by ion-exchange and gel filtration chromatography. The proteins were tested in a series of assays to determine their biological functions. Large-scale glycan microarray analysis with 610 different glycans was used to investigate the receptor binding properties of the HEF-mut protein. The result revealed that the HEF-mut protein only binds robustly to 9-O-Ac-Sia glycan derivatives, with different relative fluorescence units (RFU) (Fig 1A, S1 Table). The structural formulas of the top four binding glycans are shown in Fig 1A. In order to further characterize the binding properties of IDV HEF, we chose to use a more extended array with broader and paired (Neu5Ac- or Neu5Gc-based, and α2–3 or α2-6-linked) 9-O-Ac and non-O-Ac-sialoglycans [36]. The result, summarized in Fig 1B and S2 Table, indicate that IDV HEF-mut bind both α2–3 and α2-6-linked 9-O-Ac-Sias. Another interesting finding was the IDV HEF-mut can tolerate differentially modifications at C5, not only bind to 5-N-Ac-Sias, but also 5-N-Gc-Sias (Fig 1B). We also tried to perform the glycan array analysis of ICV HEF-mut protein, and to our surprise, the ICV HEF-mut protein did not bind to the glycan array with synthetic short glycans at all, which might be due to the lower binding affinity, and/or relative instability of the mutated protein.

**Fig 2:**
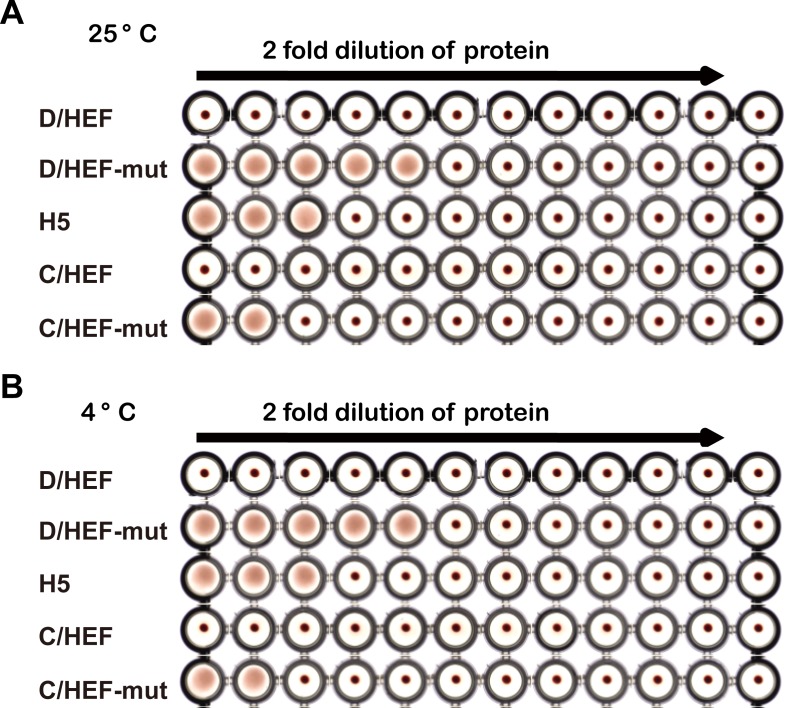
Hemagglutination assay of the IDV and ICV HEF proteins at 25°C or 4°C. Receptor binding activity of HEF protein was assessed by hemagglutination assay with chicken erythrocytes. Serial dilutions of purified HEF proteins (100 μg/mL to 0.1 μg/mL per well) were mixed with washed chicken erythrocytes and incubated to analyze the receptor binding and cross-linking of chicken erythrocytes at two different temperatures 25°C (A) or 4°C (B). The H5 HA protein was used as positive control. Positive hemagglutination results formed a uniform reddish color across the well, whereas negative results appeared as dots in the center of round-bottomed plates due to erythrocytes sedimentation.

**Fig 3:**
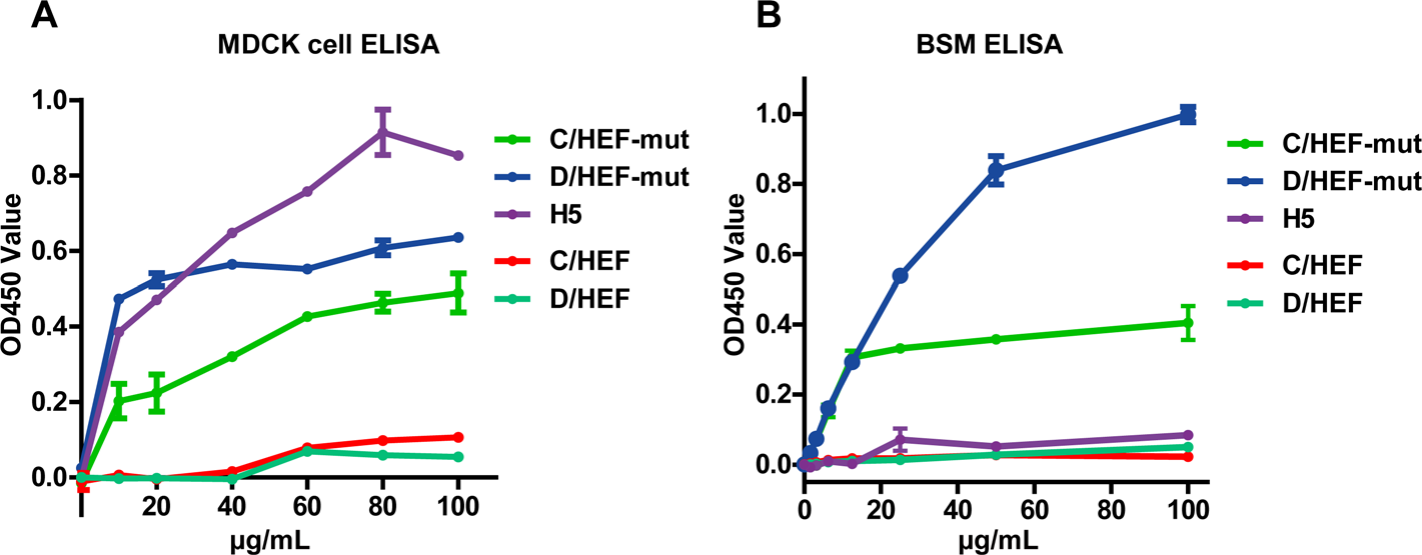
Receptor binding of the IDV and ICV HEF proteins using MDCK cell based ELISA or solid-phase lectin-binding assay towards BSM. (A) Receptor binding activity of HEF protein was assayed by MDCK cell based ELISA. After MDCK cell were fixed and blocked, different concentrations of His-tagged HEF or H5 HA proteins (10 μg/mL, 20 μg/mL, 40 μg/mL, 60 μg/mL, 80 μg/mL, 100 μg/mL) was incubated for detection by ELISA method. (B) Binding of two-fold serial dilutions (starting at 100 μg/mL) of HEF protein was assayed in a solid-phase lectin-binding assay towards BSM. Mean values were determined from three duplicates and are presented with SDs indicated by error bars.

In addition, both the IDV and ICV HEF-mut proteins, but not HEF, display the hemagglutination activities (Fig 2) and specifically binding activities to the Madin-Darby canine kidney (MDCK) cells and bovine submaxillary mucins (BSM) which are enriched in 9-Ac Sias (Fig 3A and 3B). More importantly, the IDV HEF-mut displays much stronger binding capacity than the ICV HEF-mut, which is compatible with our glycan array analysis.

### IDV HEF has esterase enzyme activity

**Fig 4:**
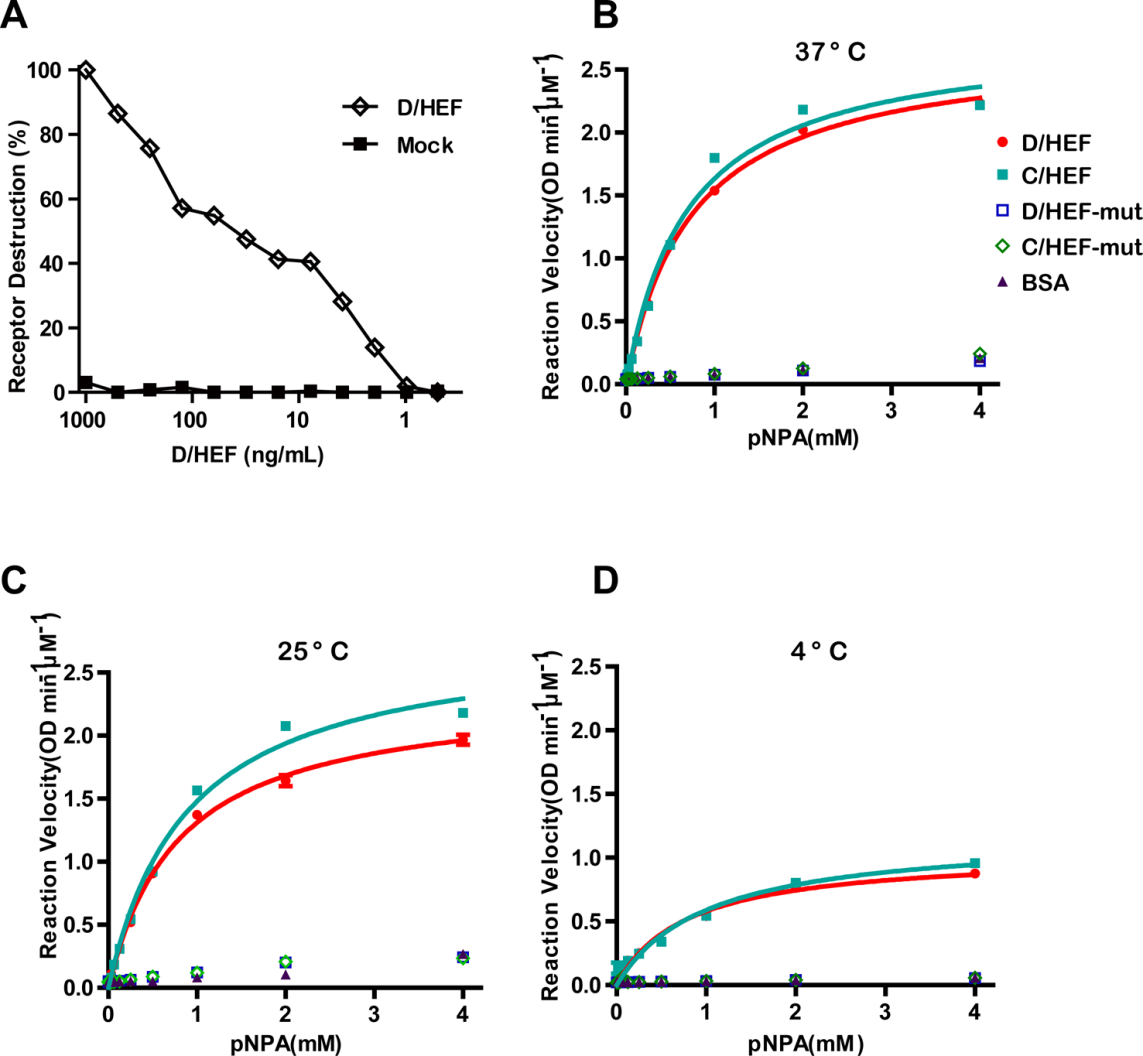
Esterase activity assay of HEF proteins. (A) Receptor destroying enzyme activity towards BSM. Coated BSM was treated with two-fold serial dilutions (starting at 100 ng/mL) of IDV enzymatically-active HEF proteins for 1 h at 37°C and 9-O-Ac-Sia content was detected by solid-phase lectin-binding assay with IDV HEF-mut protein. Decrease in signal as compared to untreated BSM is plotted in percentages. (B-D) Esterase kinetic analysis of HEF proteins against pNPA at 37°C (B), 25°C (C) and 4°C (D). The reaction velocity (OD min^-1^μM^-1^ protein) is shown based on substrate conversion. PNPA substrate was used at a final concentration of 0–4mM. Both IDV(red cycle) and ICV HEF (cyan square) display obvious enzymatic activities, whereas IDV (green rhombus) and ICV HEF-mut (blue square) lack sialidase activity. BSA (magenta triangle) was used as a negative control. Mean values were determined from three duplicates and are presented with SDs indicated by error bars.

HEF protein was further tested for its enzymatic activity using two substrates, BSM and p-nitrophenyl acetate (pNPA). Firstly, we found that the receptors in BSM could be destroyed by treatment with HEF protein (Fig 4A). Secondly, we performed an enzyme kinetics assay using pNPA as a substrate at three different temperatures (37°C, 25°C and 4°C). Both IDV and ICV HEF displayed obvious esterase activities while HEF-mut proteins exhibited no enzymatic activities (Fig 4B–4D). With the decrease of temperature, the esterase activities of both IDV and ICV HEF decreased as well. However, both IDV and ICV HEF still retain noticeable esterase activities even at 4°C (Fig 4D). This may explain why wild type HEF proteins do not show either hemagglutination activity or receptor binding activities clearly (Figs 2 and 3), as the receptors are being destroyed by their esterase activities, even at 4°C.

### Host tissue tropism of IDV and ICV HEF

**Fig 5:**
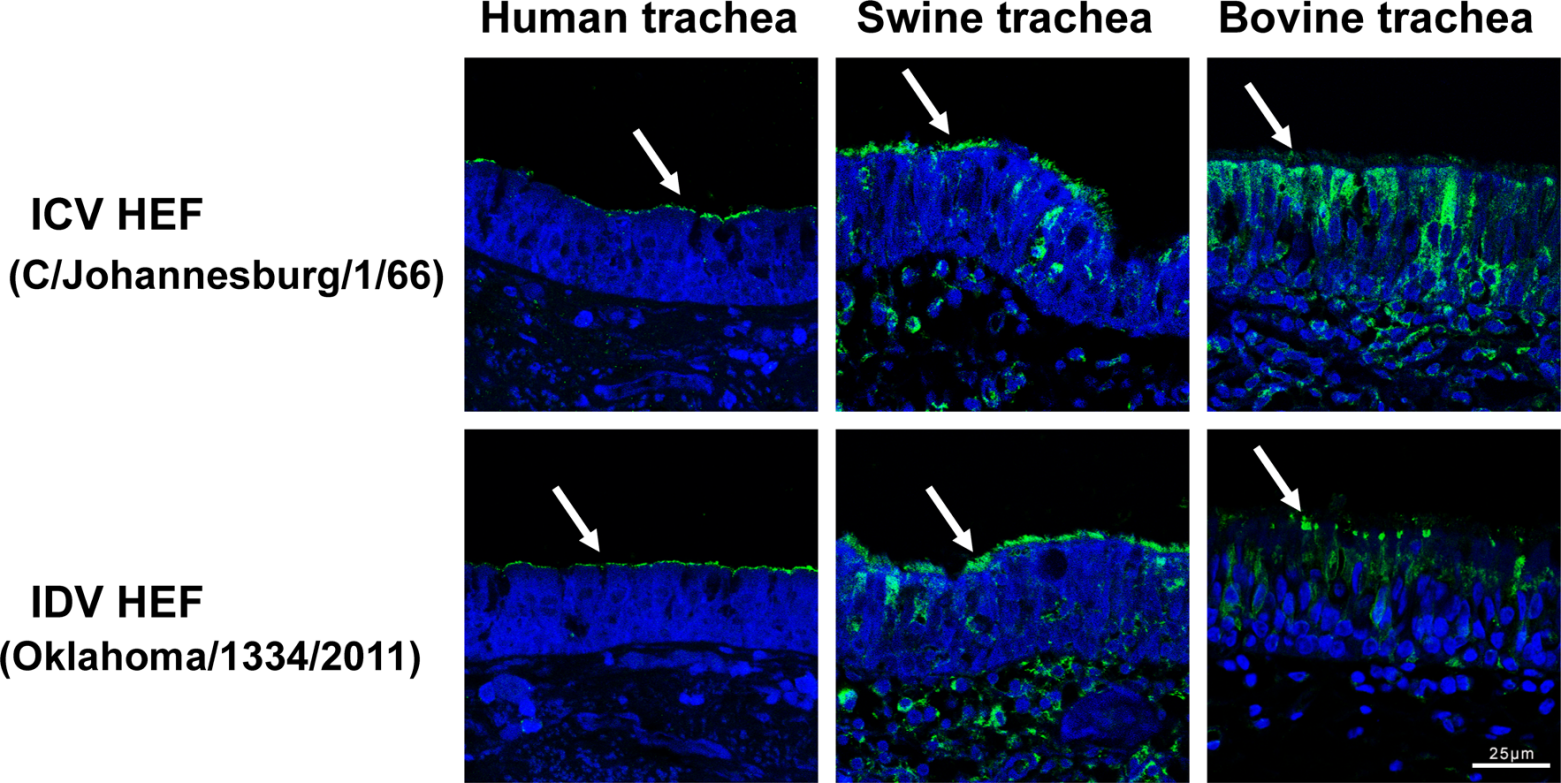
Staining of human, swine and bovine trachea with either ICV or IDV HEF protein. Paraffinized tissue sections were stained with recombinant HEF-mut protein derived from the baculovirus expression system. Specific staining by recombinant protein (green) is indicated by white arrows. Nuclei were counterstained with DAPI (blue).

To examine the host tissue tropisms of IDV and ICV, we used soluble recombinant HEF-mut proteins of these two viruses to stain paraffinized human, swine or bovine trachea sections. Interestingly, the apical surfaces of the human, swine and bovine trachea showed positive staining with ICV HEF-mut and IDV HEF-mut (Fig 5), indicating the apical surfaces of the trachea of these three species all exhibit the receptor 9-O-Ac-Sia. Moreover, the tracheas of swine and bovine display brighter staining than that of the human trachea (Fig 5).

### Overall structure

**Fig 6:**
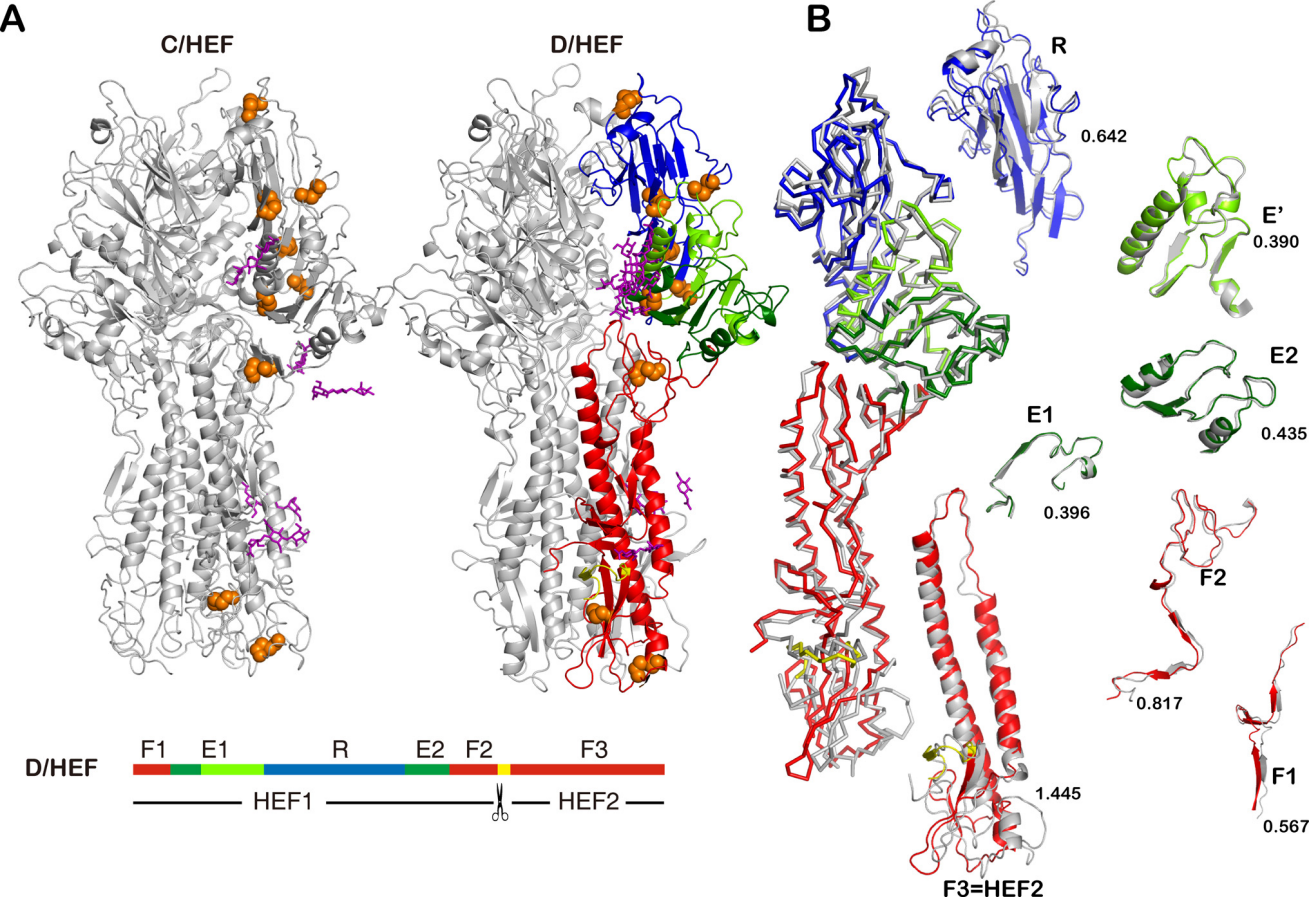
Overall crystal structure of the IDV HEF protein. (A) Ribbon representation of the trimers of ICV and IDV HEF. For ICV HEF (PDB code: 1FLC), the trimer is colored gray; For IDV HEF, one HEF protomer is colored by domain separately: fusion domain (F1, F2, F3, red), receptor domain (R, blue) and esterase domain (E1, E', E2, green) and the others are colored gray. Fusion peptides are shown in yellow. N-linked glycans are highlighted in sticks (magenta) and disulfide linkages are shown in orange. Linear order of the sequence segments in HEF colored by domains. The scissor indicates the HEF cleavage site. (B) Superposition of IDV HEF and ICV HEF subdomains are shown with RMSD values and colored by domains.

In order to study the molecular basis of IDV HEF and its receptor / substrate binding mode we solved the native X-ray crystallography structures of wild type and mutant HEFs both at a resolution of 2.4 Å (Table 1). A Dali search within the Protein Data Bank (PDB) revealed that the IDV HEF structure most resembles the ICV HEF structure which has two subunits HEF1 and HEF2 (PDB code, 1FLC; Z score, 53.5 for HEF1 and 9.0 for HEF2) [35]. Following the initial domain nomenclature used in the description of the structure of ICV HEF, we divided the IDV HEF structure into three domains: receptor binding domain (R), esterase domain (consisting of E1, E' and E2 subdomains) and fusion domain (consisting of F1, F2 and F3 subdomains). Both the overall structure (Fig 6A) and the structural folds of individual subdomains (Fig 6B) of IDV HEF and ICV HEF are remarkably similar. The E domains are the most conserved regions and display root mean square differences (RMSDs) on main chain C atoms at 0.396 Å, 0.390 Å and 0.435 Å for E1, E' and E2, respectively, with corresponding sequence identities of 66.7%, 68.8% and 56.6%, respectively. A comparison of R domains reveals a RMSD of 0.642 Å and a sequence identity of 46.3%. The F1 and F2 subdomains share 42.1% and 41.2% sequence identities and display RMSDs of 0.567 Å and 0.817 Å, respectively. Notably, the F3 subdomain shares 56.8% sequence identity but the RMSD reaches 1.445 Å. The F3 subdomain contains the fusion peptide, which is important for the viral membrane fusion.

**Table 1:** Crystallographic data collection and refinement statistics.

	IDV-HEF	IDV-HEF-mut	IDV-HEF-mut with 9-O-Ac-3'SLN	IDV-HEF-mut with 9-O-Ac-3'Sle^C^	IDV-HEF with 9-N-Ac-Sia
**Data collection**					
Space group	P21 3	P21 3	P21 3	P21 3	I21 3
Cell dimensions					
*a*, *b*, *c* (Å)	165.22, 165.22, 165.22	164.335, 164.335, 164.335	164.897, 164.897, 164.897	164.825, 164.825, 164.825	165.30, 165.30, 165.30
α, β, γ(°)	90, 90, 90	90, 90, 90	90, 90, 90	90, 90, 90	90, 90, 90
Resolution (Å)	50.00–2.40 (2.49–2.40)	50.00–2.40 (2.49–2.40)	50.00–2.20 (2.28–2.20)	50.00–2.20 (2.28–2.20)	50.00–3.10 (3.21–3.10)
*R*_merge_	0.114 (0.524)	0.121 (0.769)	0.092 (0.596)	0.093 (0.565)	0.079 (0.858)
*I* / δ*I* ^*a*^	19.8 (4.9)	15.5 (2.6)	24.5(3.4)	25.2 (5.0)	23.30 (2.40)
Completeness (%)	100.0 (100.0)	99.4 (100.0)	99.6(96.5)	100.0 (100.0)	99.9 (100.00)
Redundancy	8.6 (8.7)	6.8 (6.8)	9.2 (7.3)	9.7 (9.7)	7.2 (7.4)
**Refinement**					
Resolution (Å)	47.7–2.40	49.55–2.40	47.60–2.20	47.71–2.20	38.96–3.10
No. reflections	58686	57383	75424	75303	13867
*R*_work_ / *R*_free_	0.2010/0.2447	0.2046/0.2533	0.2035/0.2428	0.2045/0.2442	0.2244/0.2681
No. atoms					
Protein	9071	8700	9084	9082	4547
Ligand/ion	16	0	70	100	28
Water	566	544	675	629	0
*B*-factors					
Protein	44.20	43.77	44.10	48.26	118.90
Ligand/ion	46.10	-	78.65	94.25	123.80
Water	42.60	41.00	40.00	43.66	-
R.m.s. deviations					
Bond lengths (Å)	0.006	0.018	0.006	0.010	0.011
Bond angles (°)	1.299	1.488	1.005	1.501	1.478
Ramachandran analysis					
Most favoured (%)	86.3	84.6	86.6	86.6	78.3
Additional allowed (%)	11.6	13.5	11.8	11.8	20.1
Generally allowed (%)	1.9	1.8	1.3	1.3	1.0
Disallowed (%)	0.3	0.2	0.2	0.2	0.6

*Values in parentheses are for highest-resolution shell.

### The receptor-binding site of IDV HEF

**Fig 7:**
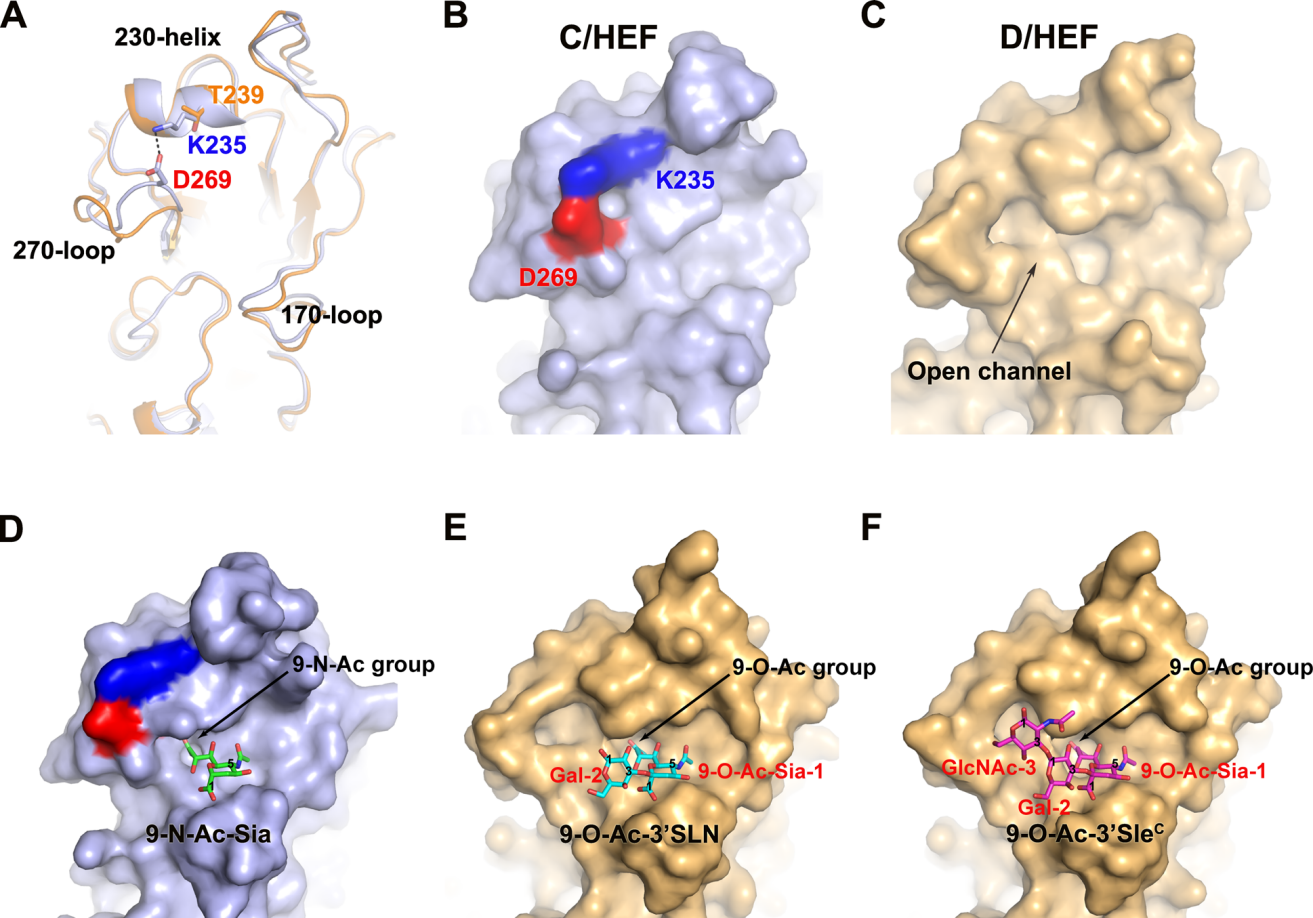
The variable receptor-binding site in the IDV HEF. (A) Comparison of the receptor-binding sites of ICV (light blue) and IDV (orange) HEFs. A salt bridge interaction is formed between the cationic K235 (blue) of 230-helix and the anionic D269 (red) of 270-loop, shown in a dashed line. (B and C) The surface presentations of the salt bridge interaction in ICV HEF (B) and absence of the salt bridge in IDV HEF (C), resulting in an open channel. (D-F) The surface presentations of ICV HEF binding to 9-N-Ac-Sia (D) and IDV HEF-mut binding to 9-O-Ac-3'SLN (E) or 9-O-Ac-3'Sle^C^ (F).

**Fig 8:**
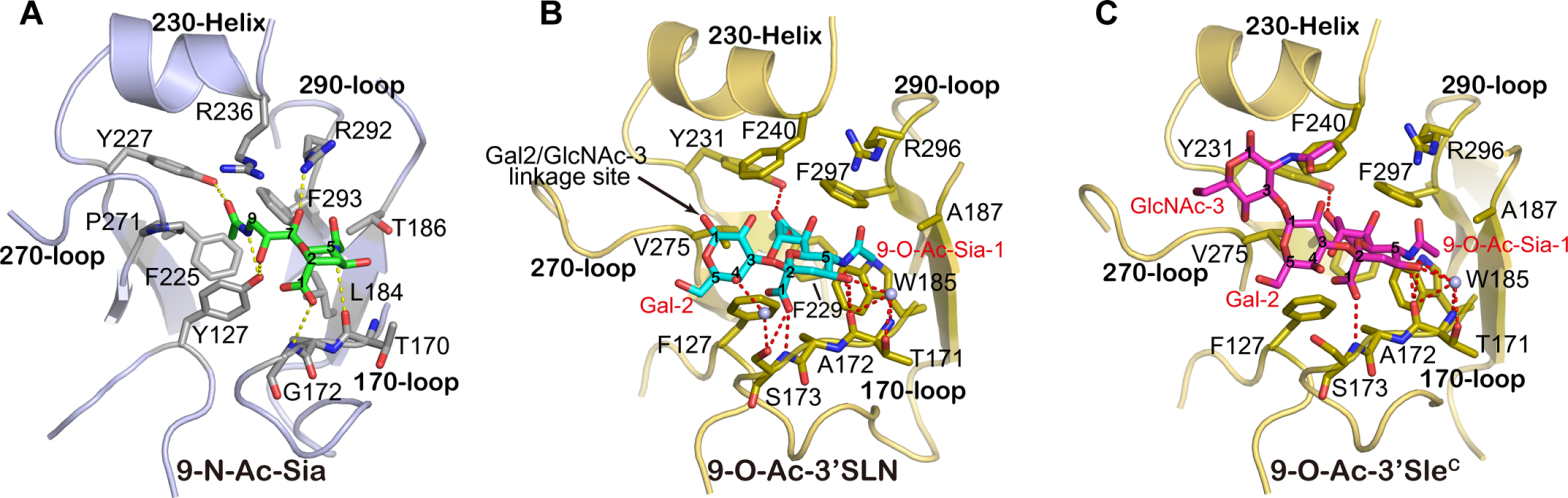
Detailed Interactions of the Receptor-Binding Site in the IDV HEF. Molecular interactions of the ICV HEF binding to 9-N-Ac-Sia (A) and IDV HEF-mut binding to 9-O-Ac-3'SLN (B) or 9-O-Ac-3'Sle^C^ (C). The secondary elements of the HEF receptor-binding site (170-loop, 230-helix, 270-loop and 290-loop) are labeled and shown in cartoon representation. Selected residues and receptor analogs are labeled and shown in sticks. The water molecules mediating hydrogen-bonding interactions are shown as spheres.

To understand the molecular basis of IDV HEF receptor binding, the HEF-mut crystals] were soaked with glycans 9-O-Ac-3'Sle^C^ (Tr323, Neu5,9Ac_2_α2-3Galβ1-3GlcNAcβ-Sp0) and 9-O-Ac-3'SLN (Tr322, Neu5,9Ac_2_ α2-3Gal β1-4GlcNAcβ-Sp0) [(kindly provided by the Consortium for Functional Glycomics (CFG, Scripps Research Institutes, Department of Molecular Biology, La Jolla, CA) to determine the structures of the respective receptor complexes, which were resolved at a resolution of 2.2 Å (Table 1). Similar to ICV HEF, the receptor-binding site of IDV HEF is located near the top of the HEF1 globular head in a shallow cavity, surrounded by residues from four secondary structure elements: the 170-loop, 190-loop, 230-helix and 270-loop. Five base residues F127 (C HEF: Y127), W185 (C HEF: L184), Y231 (C HEF: Y227), F229 (C HEF: F225) and F297 (C HEF: F293) form the bottom of the cavity (Figs 7 and 8). However, one unique feature in the IDV HEF receptor-binding site observed is an open channel between the 230-helix and 270-loop. In ICV HEF, the positively-charged residue K235 of 230-helix and the negatively-charged residue D269 of 270-loop form a salt bridge interaction, pulling the 270-loop up to connect with 230-helix and close the channel observed in IDV HEF. Whereas for IDV HEF, at equivalent positions, T239 and A273 cannot form salt bridge interaction.

For the IDV HEF-mut/receptor complex structures, interpretable electron density is observed for all the three glycan rings of 9-O-Ac-3'Sle^C^, including Neu5,9Ac_2_ (9-O-Ac-Sia-1), galactose-2 (Gal-2), and N-acetylglucosamine-3 (GlcNAc-3); while for 9-O-Ac-3'SLN, electron density for the first two sugars is well defined (Figs 7 and S1). The glycan rings of 9-O-Ac-3'Sle^C^ go through the open channel between the 230-helix and 270-loop (Fig 7F). The open channel in the receptor-binding cavity of IDV could provide a structural basis for accommodating different receptors with diverse glycan rings in different cell types, which should be tested in the future.

Compared with the ICV HEF receptor complex, the 9-O-Ac-Sia-1 of the receptor bound to IDV HEF displays a very similar orientation (Figs 7 and 8) [35]. The 9-O-acetyl group of the receptor docks into a nonpolar, hydrophobic pocket, formed by V275, F229, F240 and F297 (Fig 8). The acetyl carbonyl oxygen forms a hydrogen bond with the hydroxyl group of Y231 (ICV HEF: Y227). Y231 is highly conserved both in ICV and IDV HEF (See alignment in S2 Fig). Importantly, the conserved amino acid Y127 (Y98 in IAV HA, H3 numbering) both in ICV HEF and IAV HA has been changed to F127 in IDV HEF, which is the same as IBV HA [37]. The absence of two hydrogen-bonding interactions between hydroxyl group of Y127 and the 8-hydroxyl and 9-amide of the ligand, pushes the 9-O-acetyl moiety of 9-O-Ac-sia-1 to the other side by ~1.1 Å. In exchange, two more hydrogen-bonding interactions between the carbonyl group of C4 and T171 mediated by water stabilize the sugar ring conformation and prevent its excessive shift and rotation. The 5-N-acetyl group of the ligand fits into a hydrophobic pocket formed mainly by W185, and forms a hydrogen-bonding interaction with A172. In addition, the carboxyl group of C1 formed two hydrogen-bonding interactions with S173.

Both 9-O-Ac-3'Sle^C^ and 9-O-Ac-3'SLN bind in a *cis* conformation (Figs 7 and 8), similar to the IAV avian SH-H7N9 HA binding with avian-like (α2–3) receptor structures [38]. But the majority of the interactions between ligands and IDV HEF are made by the 9-O-Ac-Sia-1 moiety, whereas the glycan portion of the ligands do not make significant contacts with IDV HEF, except for a hydrogen bond between the Gal-2 of 9-O-Ac-3'SLN and S173.

### IDV HEF shares a conserved esterase pocket with ICV HEF

**Fig 9:**
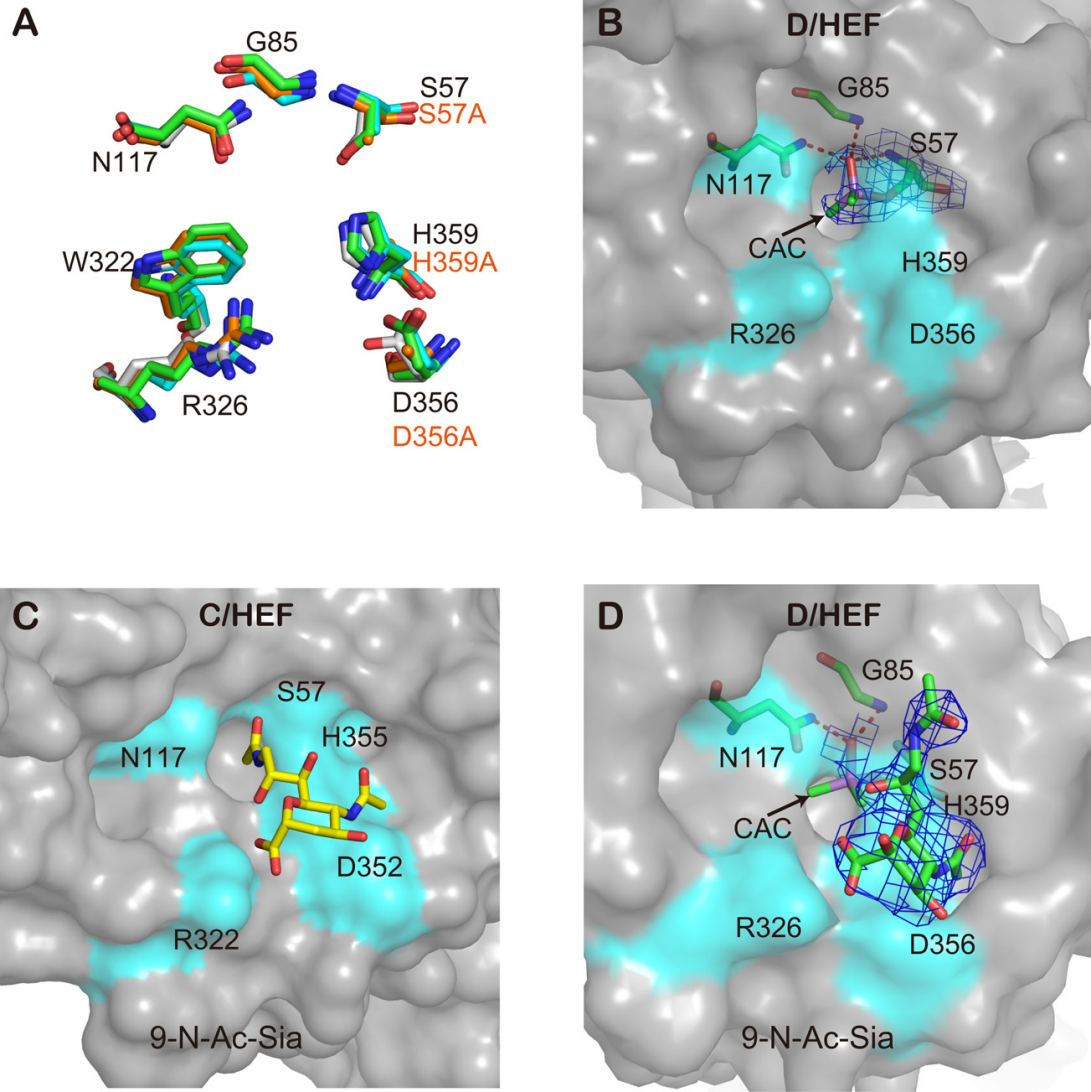
The esterase pocket of the IDV HEF. (A) Superposition of IDV HEF (green), IDV HEF-mut (orange), ICV HEF (gray) and BCoV HE (cyan) esterase sites. Residue labeling refers to IDV HEF. (B) Surface representation of the esterase pocket of IDV HEF. S57 is covalently modified by the addition of a dimethylarsenic group (cacodylate ion, CAC). The 2Fo-Fc electron density map for this dimethylarsenic modified serine contoured at 1.0 sigma is represented in blue. (C) Surface representation of ICV HEF esterase pocket with substrate analog 9-N-Ac-Sia. (D) Surface representation of IDV HEF esterase pocket with substrate analog 9-N-Ac-Sia in presence of arsenic modification of S57. The 2Fo-Fc electron density map for 9-N-Ac-Sia and the dimethylarsenic modified S57 contoured at 1.0 sigma is represented in blue.

**Fig 10:**
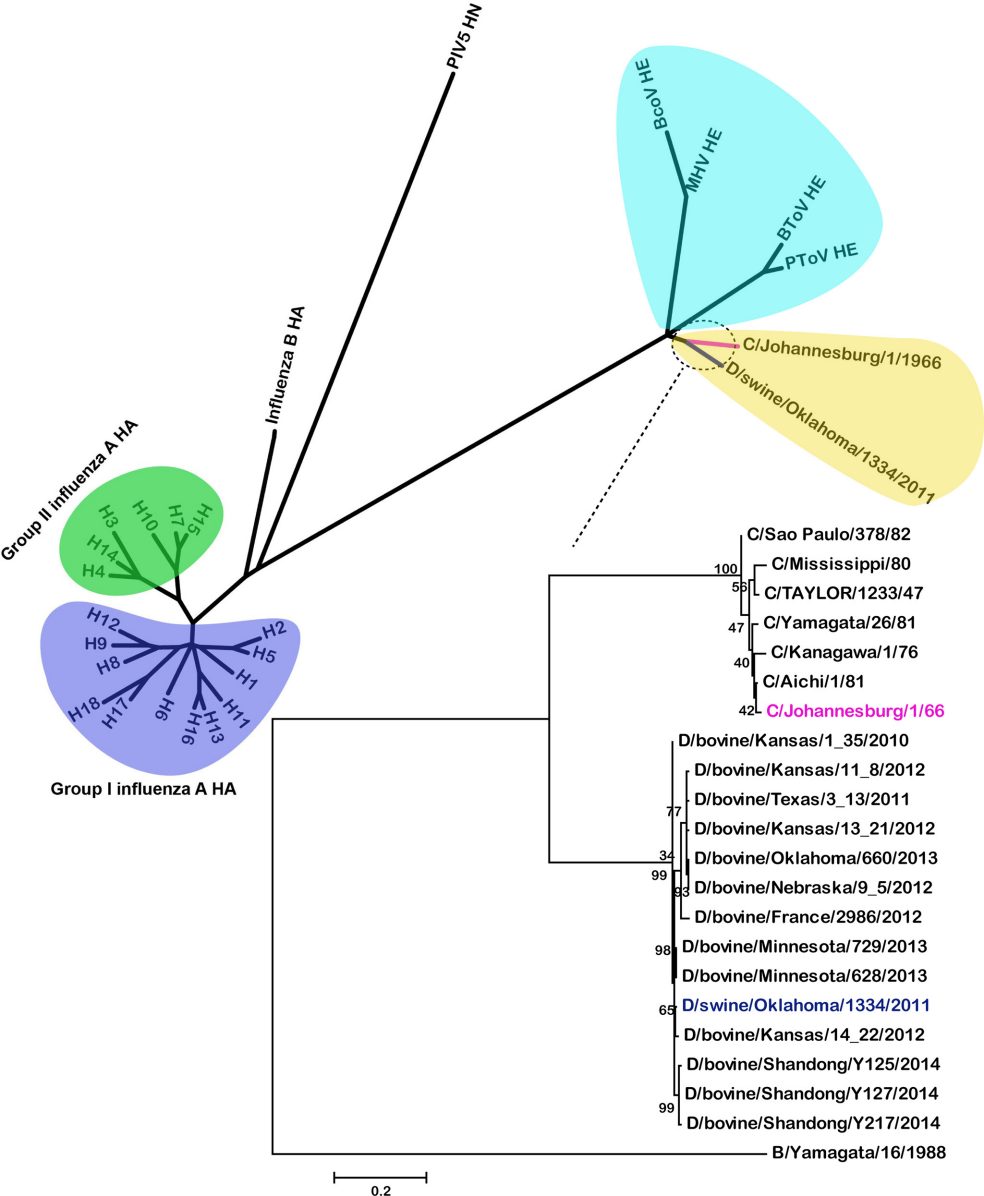
Phylogenetic trees of influenza virus HA/HEF glycoproteins and the related HE or HN proteins. Maximum-likelihood analysis in combination with 1000 bootstrap replicates was used to derive trees. Evolutionary analyses were conducted in MEGA6 [40].

The E domain of IDV HEF, harboring the receptor-destroying enzyme (RDE) activity, has a hydrolase fold that is highly similar to that of ICV HEF. The active-site architecture of the HEF sialate-9-O-acetylesterase is fully conserved in ICV and IDV HEF with S57, D356 and H359 creating a catalytic triad and with the side chain of N117 and the NH groups of S57 and G85 forming an oxyanion hole (Fig 9A) [35]. The pocket is extremely conserved not only in IDV HEF and ICV HEF, but also in some nidovirus hemagglutinin-esterase (HE) proteins such as bovine coronavirus (BCoV) HE, porcine torovirus (PToV) HE and bovine torovirus (BToV) HE [39]. Phylogenetic analysis also shows the relationship between HEF and HE to be much closer than the relationship between HEF and HA or hemagglutinin-neuraminidase (HN) proteins (Fig 10), implying common ancestral origins.

### Serine 57 is the active-site amino acid of the acetylesterase and is modified by arsenic in the structure

Due to the presence of cacodylate buffer in the crystallization conditions, S57 in our IDV HEF structure is covalently modified by the addition of a dimethylarsenic group. The covalent modification of active site serine by arsenic was first reported by Ian Wilson’s group in 2003 [41], when they solved the structure of an acetyl esterase, HerE, and its complex with the inhibitor dimethylarsinic acid, and illustrated the mechanism of the broad scope inhibition of serine hydrolases by As(V)-containing organic compounds [41]. The electron density of the covalent modification of S57 is shown in Fig 9B. This modification is only observed in the wild type HEF structure, while in HEF-mut structure, there is no such electron density due to a S57A mutation. Another serine esterase inhibitor diisopropyl fluorophosphate (DFP) is known to bind covalently to the serine in the active site of serine esterases. DFP-treated ICV bound specifically and irreversibly to cells expressing 9-O-Ac-Sias. This provided a probe for detecting 9-O-Ac-Sias [4] and S57 was demonstrated as the active-site serine in the acetylesterase of ICV [42]. DFP has also been used to determine the active site of enzymes by solving the complex crystal structure [43, 44]. In our IDV HEF complex structure with its receptor analogue 9-N-Ac-Sia, we found that the 9-N-acetyl group of the substrate was tilted up against the oxyanion hole as the covalent modification of S57 by arsenic blocked the insertion of the 9-N-acetyl group inserting into enzymatic site (Fig 9D).

### An exposed fusion peptide

**Fig 11:**
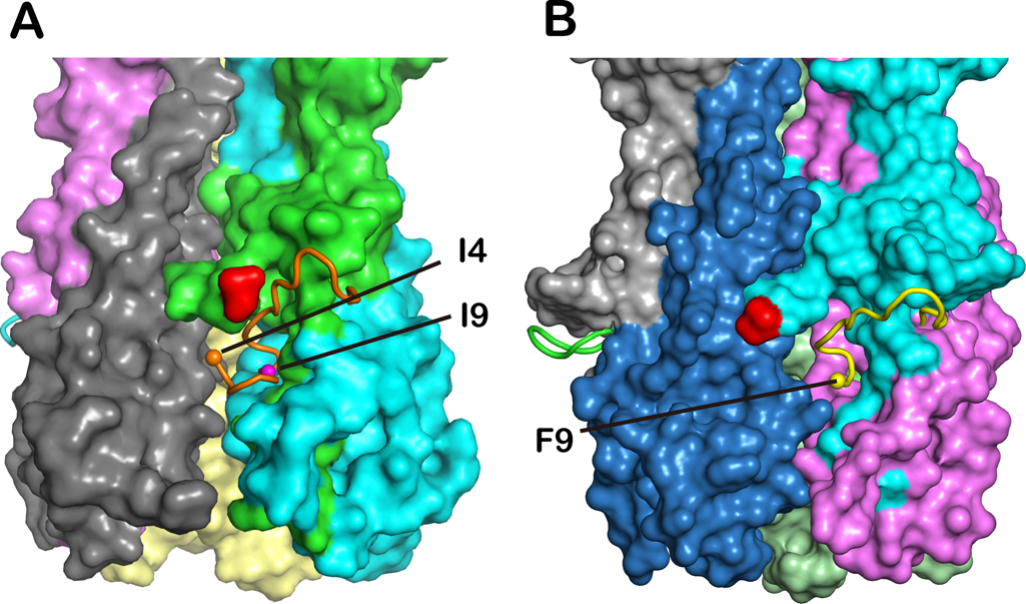
Exposed fusion peptide in the cleavage Site of IDV HEF. (A) Surface diagram of the fusion peptide in the ICV HEF structure. The fusion peptide is partially exposed away from the cavity. (B) Surface diagram of the fusion peptide in the IDV HEF structure. The fusion peptide is exposed away from the cavity.

In our IDV HEF structure, IDV HEF is proteolytically cleaved into the subunits HEF1 and HEF2. For ICV, the HEF protein contain a monobasic cleavage site located in the stem region of the trimeric spike and can be cleaved by protease hydrolyses between R432 and I433 in to HEF1 and HEF2 [35, 45]. This monobasic cleavage site was very conserved both in ICV HEF (R432) and IDV HEF (R439), and the first eight residues of fusion loop were conserved between ICV and IDV HEF sequences too (S2 Fig). To confirm the proteolytic processing, we isolated IDV HEF crystals and checked for HEF2 band both by SDS-PAGE and western blot with an antibody recognizing the hexa-His tag engineered at the C terminus (S3A Fig). Furthermore, N-terminal amino acid sequencing of the HEF2 band revealed that the first five amino acids of HEF2 were IFGID (S3B–S3F Fig), the same with ICV HEF. In all solved cleaved influenza A and B HA structures, the N-terminal HA2 fusion peptide inserts into an electronegative cavity composed of different HA protomers, except in the bat-derived H17 and H18, which display an exposed fusion peptide [46, 47]. The exposed fusion peptide has also been observed previously in the HEF protein of ICV [35] (Fig 11A) and in the cleavage site of IDV HEF structure (Fig 11B). Unambiguous electron density was seen from the ninth residue (F9) of the fusion peptide (S4 Fig). Although we cannot observe the first eight residues in the structure, different orientations of F9 in the HEF structure and other known cleaved HA structures helped to confirm that the fusion peptide does not insert into the cavity.

## Discussion

Since the first ICV strain (C/Taylor/1233/47) was isolated in 1947 during an epidemic of respiratory illness, hundreds of viruses have been isolated in clinical specimens [48–50], due to its inconspicuous or mild symptoms and lack of suitable cell lines for virus isolation [51]. Recently, a dozen of novel IDV strains have been isolated from both pigs and cattle, strains that are distantly related to human ICV [20, 21, 23, 25, 26, 52]. Ferrets, guinea pigs and small ruminants (sheep and goats) have also been found to be susceptible to the virus [20, 24, 29]. Clearly, the viral traits necessary for host switching are different between ICV and IDV.

Here, we solved the structure of IDV HEF alone and in complex with its receptor analogs. We found that the overall structure of IDV is extremely similar to that of ICV, despite sharing a relatively low sequence identity of only 53% [20]. This finding is similar to the structural similarity observed between HAs of IAV and IBV [37]. The IDV HEF structure was modeled by Modeller 9.10 using the ICV HEF as template in a previous study [20], and they had an overall similar structure with ICV HEF. However, the details of interactions between the ligands and receptor-binding site and substrate binding site were not predicted precisely. Notably, we found that the receptor-binding site of IDV HEF occurs in an open channel between 230-helix and 270-loop. By contrast, in the receptor-binding site of ICV HEF the K235 of 230-helix and D269 of 270-loop form a salt bridge interaction, pulling the 270-loop up to connect with 230-helix and close the channel. The open channel in the receptor-binding site of IDV HEF could provide the space to accommodate an array of glycan linkages found in diverse host receptors which might explain why IDV has a broad cell tropism. Although the receptor specificity and adaptability of viral surface protein is one crucial determinant for host jump, other viral proteins may also be restrictive barriers to viral host range. PA, PB1 and PB2, which comprise the RNA-dependent RNA polymerase complex have long been implicated in playing a crucial role in determining host tropism [53, 54]. Furthermore, innate immune responses, intracellular factors and cross-species contacts may also affect host adaptation [55]. Therefore, a better understanding of the ecological, evolutionary and molecular mechanisms of IDV is essential in order to explain the broader host and cellular range of IDV and accurately assess the risk of transmission to other host species.

We determined that IDV HEF uses the glycan derivatives, 9-O-Ac-Sia as its receptor. We show that both ICV and IDV HEF proteins can bind to the trachea of human, swine and bovine. Considering the ability of IDV to transmit in ferrets and guinea pigs, and its pathogenicity in pigs and cattle, its public health threat for transmission to human must be monitored. A number of studies have examined occupational risk factors for zoonotic influenza virus infections, including open bird market workers, swine workers, meat processing workers, veterinarians and poultry workers, concluding that these populations are indeed at greater risk of infection with zoonotic IAV [56, 57]. Therefore, the surveillance of IDV infection in animal farm workers with influenza-like illness must be performed. Moreover, given the major economic importance of cattle and swine, further research into the pathobiology of IDV in these hosts, especially putative role in BRDC, needs to be conducted.

Another important phenomenon observed earlier is that IDV is unable to reassort with the ICV [20, 21]. As the only glycoprotein on the virus surface, it was suggested that the HEF protein may affect virus reassortment by incompatible protein functions in ICV and IDV [20, 21]. However, herein we have described the structure of IDV HEF and found it to be highly structurally and functionally similar to ICV HEF. Previous work has shown that there exist two discrepancies at the extremely conserved non-coding regions of seven RNA segments (the first twelve nucleotides at each 3' end as well as the last eleven nucleotides at each 5' end) between ICV and IDV genomes [20]. In addition, the non-conserved non-coding regions adjacent to each coding region are significantly variable [20]. Therefore, we propose that the packaging signal compatibility may be the important factor for the heterotypic incompatibilities between ICV and IDV.

The esterase pocket of IDV HEF is highly conserved among ICV and nidovirus HE proteins and could represent a potential drug target for developing broad-spectrum inhibitors. Therefore, rational modification of the substrate analog based on our structures may provide a potential route for the development of novel therapeutics against both orthomyxovirus HEFs and nidovirus HEs.

In conclusion, our functional and structural approach for the IDV surface protein HEF clearly reveals the virus’ similarities and distinctions in comparison to the ICV and that the virus could be a potential concern for public health as the viral HEF can bind human trachea epithelia.

## Materials and Methods

### Ethics statement

Paraffin-embedded normal human tracheal tissue sections were purchased from Auragene Bioscience (China). Formalin-fixed normal swine (5 months old domestic pig) and bovine (8 months old cattle) tracheal tissues were obtained from Zhongmu institutes of China animal husbandry industry with approval.

### Cell culture

Madin-Darby canine kidney (MDCK) cells (NBL2, obtained from cell resource center of Shanghai Institutes for Biological Sciences, Chinese Academy of Sciences) were cultured in Dulbecco's modified Eagle's medium (DMEM, Gibco) supplemented with 10% fetal bovine serum in a humidified chamber containing 5% CO_2_ at 37°C.

### Gene cloning, protein expression and purification

The gene of the HEF from D/OK strain (NCBI accession no. JQ922308) encoding the ectodomains (amino acid residues 3–605 after deletion of the signal peptide) was cloned into the baculovirus transfer vector pFastBac1 (Invitrogen) in-frame with an N-terminal gp67 signal peptide for secretion and a His_6_-tag at the C terminus for purification [31, 32]. To allow expression of an enzymatically inactive HEF protein (HEF-mut), the codons for the esterase catalytic residues S57, D356 and H359 were all substituted by Ala by site directed mutagenesis using the overlap extension PCR method and inserted to pFastBac1 in the same way. Recombinant pFastBac1 plasmid was used to transform DH10Bac *Escherichia coli* (Invitrogen). Transfection and virus amplification were performed according to the Bac-to-Bac baculovirus expression system manual (Invitrogen) [30, 33, 58]. HEF proteins were produced by infecting suspension cultures of Hi5 cells (Invitrogen) for 2 days. Soluble HEFs were recovered from cell supernatants by metal affinity chromatography using a HisTrap HP 5 ml column (GE Healthcare), then purified by ion-exchange chromatography using a RESOURCE Q 6 ml column (GE Healthcare). For crystallization, the proteins were further purified by gel filtration chromatography using a Superdex 200 10/300 GL column (GE Healthcare) with a running buffer of 20 mM Tris–HCl and 150 mM NaCl (pH 8.0), and the collected protein fractions were concentrated to 10 mg/mL using a membrane concentrator with a molecular weight cutoff of 10 kDa (Millipore). Both the wild type and enzymatically inactive (with S57A, D356A and H359A mutations) ICV HEF (C/Johannesburg/1/66) protein (NCBI accession no.AM410041, amino acid residues 1–597 after deletion of the signal peptide) were expressed and purified in the same as that of HEF-D/OK. HA of A/Anhui/1/2005 (H5N1) was prepared as described in our previous report [38].

### Crystallization, data collection and structure determination

The initial screening trials were set up with commercial crystallization kits (Molecular Dimensions) using the sitting drop vapor diffusion method. Normally, 1 μL protein was mixed with 1 μL reservoir solution. The resultant drop was then sealed, equilibrating against 100 μL reservoir solution at 4 or 18°C. After optimization and seeding, diffractable crystals were obtained in a reservoir solution of 0.1 M PCTP (Propionic acid, Cacodylate, Bis-tris propane system) buffer pH 8.5, 22.5% w/v PEG 1500 for both HEF and HEF-mut protein at 4°C. For receptor complexes, HEF-mut crystals were soaked in a reservoir solution containing 10 mM 9-O-Ac-3'Sle^C^ or 9-O-Ac-3'SLN at 4°C for 5 hr. For receptor analog complexes, HEF crystals were soaked in a reservoir solution containing 8 mM N -Acetyl-9-(acetylamino)-9-deoxyneuraminic Acid (or 9-N-Ac-Sia, TRC, Canada) at 4°C for 5 hr. All crystals were flash-cooled in liquid nitrogen after a brief soaking in reservoir solution with the addition of 17% w/v PEG 1500. The X-ray diffraction data were collected at Shanghai Synchrotron Radiation Facility (SSRF) beamline 17U, with a wavelength of 1.000 angstrom, at a temperature of 100K. All data were processed with HKL2000 software [59].

The HEF structures were solved by the molecular replacement (MR) method using Phaser [60] from the CCP4 program suite [61], with the structure of human ICV HEF (PDB: 1FLC) as the search model. Model building and refinement were performed using the COOT [62] and REFMAC5 [63] programs, respectively. The HEF receptor analog complexes were subsequently determined using the refined HEF structure as the input model. The receptor analogs were manually built using COOT based on the simulated anealing omit Fo-Fc maps and were further refined by PHENIX [64]. The stereochemical quality of the final models was assessed with the program PROCHECK [65]. Final statistics for data collection and structure refinement is represented in Table 1.

### Glycan microarray

The microarray analysis was first performed by applying the IDV HEF-mut protein to the array at 200 μg/mL and detecting with a His antibody labeled with Alexa488. The experiments were performed in replicates of six at CFG using a version 5.1 CFG array consisting of 610 glycans. The highest and lowest points from each set of six replicates were removed, so the average is of four values rather than six. This eliminates some of the false hits that contain a single very high or low point.

Then, we chose to use a more specific array with broader and paired (Neu5Ac- or Neu5Gc-based, and α2–3 or α2-6-linked) 9-O-Ac and non-O-Ac-sialoglycans to further characterize the binding properties of IDV and ICV HEF [36]. Glycan microarrays were fabricated using epoxide-derivatized slides as previously described [36]. Printed glycan microarray slides were blocked by ethanolamine, washed and dried. Slides were then fitted in a multi-well microarray hybridization cassette (AHC4X8S, Arrayit, Sunnyvale, CA, USA) to divide into 8 subarrays. The subarrays were blocked with Ovalbumin (1% w/v) in PBS (pH 7.4) for 1 hr at room temperature (RT), with gentle shaking. Subsequently, the blocking solution was removed and diluted IDV-HEF-mut and ICV-HEF-mut protein samples with 160 μg/mL were added to each subarray. After incubating the samples for 2 hr at RT with gentle shaking, the slides were washed. Diluted anti-His-HiLyte Flour 555 antibody (LifeSpan BioSciences) in PBS was added to the subarrays, incubated for 1 h at RT, washed and dried. The microarray slides were scanned by Genepix 4000B microarray scanner (Molecular Devices Corp., Union City, CA, USA). Data analysis was performed using Genepix Pro 7.0 analysis software (Molecular Devices Corp., Union City, CA). Heat map was generated according to the method previously described [36]. Ranked binding of IDV-HEF-mut and ICV-HEF-mut on the array. Binding was ranked as (glycan RFU/ maximum glycan RFU)*100. Blue and white represent the maximum and minimum, respectively.

### MDCK cell binding assays

The cell binding assays were performed in 96-well plates as previously described [46]. When the density of the MDCK cells in the wells reached 90% coverage, the plate was washed with PBS twice and fixed with ice-cold 100% methanol for 20 min. After PBST buffer (PBS with 0.05% Tween-20) washing for three times, the wells were blocked with blocking buffer [PBS, 0.05% Tween-20, 4% bovine serum albumin (BSA)]. His-tagged HEF or H5 HA protein (10 μg/mL, 20 μg/mL, 40 μg/mL, 60 μg/mL, 80 μg/mL, 100 μg/mL) was then added to each well and each concentration was performed in triplicates wells. After incubation at 37°C for 1 hr, the plate was washed three times with PBST buffer. Mouse anti-His antibody (MBL, Japan) was added to each well at a 1:1000 dilution and the plate was incubated for 1 hr. Then, the plate was washed and incubated at 37°C for 45 min with HRP-conjugated goat anti-mouse antibody (Santa Cruz, USA) at a dilution of 1:2000. Peroxidase activity was detected using TMB and the reaction was stopped by adding 2M H_2_SO_4_. Absorbance was measured at an optical density of 450 nm.

### Hemagglutination assay

Hemagglutination assay was performed in U-bottom 96-well microtest plates (Becton Dickinson, USA) according to the method previously described [66–68]. Briefly, two-fold serial dilutions in 25 μl PBS of purified HEF or HEF-mut protein (100 μg/mL to 0.1 μg/mL per well) mixed with 25 μl of a 0.5% chicken erythrocytes suspension and incubated for 1hr at 25°C or 2 hr at 4°C. Then the hemagglutination effects were observed and the plates were screened by CTL-ImmunoSpot S5 Versa Analyzer (Cellular Technology, USA).

### Solid-phase lectin binding assay (SLBA)

SLBA was performed as previously described [69]. Briefly, Corning 96 well EIA/RIA plates were coated for 16 hr at 4°C with BSM (60 μg/mL in PBS; Abnova) at 100 μL per well. The wells were washed with PBST and treated with blocking buffer for 1 hr at RT. Twofold serial dilutions of proteins containing a C-terminal His_6_ tag were prepared in blocking buffer (starting concentration 100 μg/mL) and 100 μL samples of these dilutions were added to the glycoconjugate-coated wells. Incubation was continued for 1 hr after which unbound protein was removed by washing five times. Then the wells were incubated with mouse anti-His antibody (1:1000), washed five times with washing buffer, incubated with HRP-conjugated goat anti-mouse IgG antibody (1:2000), and washed five times. Finally, the bound proteins were detected using TMB, and the reaction was stopped with 2M H_2_SO_4_. The absorbance of the resulting yellow color was read at 450 nm. To assess the enzymatic activities of IDV HEF protein towards 9-O-Ac-Sias, BSM coated plates were treated with samples from two-fold serial dilutions of IDV HEF protein (starting at 1 μg/mL in PBS, 100 μL/well) for 1 hr at 37°C. The destruction of 9-O-Ac-Sia receptor determinants was determined by SLBA with IDV HEF-mut protein (50 μg/mL in blocking buffer) as described above.

### Enzymatic activity assay

The activities of purified HEF and HEF-mut were tested using p-nitrophenyl acetate (pNPA, Sigma–Aldrich) as a substrate [70]. Proteins (50 μL) were diluted to 12.5 ng/mL using PBS buffer in each well of a 96-well plate, after which the plate was incubated at different temperatures (37°C, 25°C and 4°C). Twofold serial dilutions (0–8 mM) of preheated pNPA (50 μL) were then added at corresponding temperatures. The absorbance at 405 nm was measured immediately in a spectrophotometer every 30 seconds for 1 hr at corresponding temperatures on a microplate reader (SpectraMax M5; Molecular Devices). All assays were performed in triplicate, and the Km and Vm value for HEF were calculated using GraphPad Prism.

### Binding of HEF to human, swine and bovine trachea

Immunofluorescence assays were performed as described previously with slight modifications [71, 72]. Briefly, paraffinized human, swine or bovine trachea tissue sections were deparaffinized, rehydrated and incubated with 2% BSA in PBS for 30 min at RT to prevent nonspecific binding. Purified HEF protein was precomplexed with primary antibody (mouse anti-His-tag, MBL) and secondary antibody (Alexa Fluor 488 goat anti-mouse IgG, Invitrogen) in a molar ratio of 4:2:1, respectively, for 20 min on ice. The tissue binding was performed using precomplexed stock HEF (50 μg/ mL) in 1% BSA–PBS. Tissue sections were then incubated with the HEF–antibody complexes for 3 hr at RT. Sections were counterstained with 4', 6-diamidino-2-phenylindole (DAPI) (Beyotime; 1:2,000 in PBS) for nuclei for 20 min at RT. After thorough washing, the tissue sections were mounted and then examined by using Leica TCS SP8 laser scanning confocal microscopy.

### Western blot and N-terminal sequencing

The IDV HEF crystal samples were applied to SDS-PAGE and subsequently transferred to polyvinylidene fluoride (PVDF) membranes at 50 V for 1 hr. For western blot, the proteins were identified with a mouse monoclonal antibody of Anti-His-tag-HRP-DirecT (MBL, Japan) and a Super Signal West Pico Chemiluminescent Substrate (Thermo, USA). For N-terminal sequencing, the PVDF blot membrane was stained for 30s-50s in coomassie brilliant blue (CBB) R250 staining solution (0.1% CBB R250, 1% acetic acid, 40% methanol in Milli-Q water) and destained with destaining solution (50% methanol in Milli-Q water) under visual control until protein bands were well visible. The PVDF membrane was dried and bands of interest were cut for the N-terminal sequencing with the Edman degradation method using PROCISE491 (America Applied Biosystems).

### Accession numbers

Atomic coordinates and structure factors have been deposited in the Protein Data Bank under accession codes 5E64 for IDV HEF in native state and 5E66 in complex with 9-N-Ac-Sia, and 5E5W, 5E65, 5E62 for IDV HEF-mut and complexes with 9-O-Ac-3'SLN and 9-O-Ac-3'Sle^C^, respectively.

## Supporting Information

S1 Fig Electron density of the receptors. The panels show portions of 2Fo-Fc electron density maps for these glycan receptor analogs 9-O-Ac-3'SLN (cyan) (A) or 9-O-A-c3'Sle^C^ (magenta) (B) binding to IDV HEF-mut protein (orange, cartoon) and 9-N-Ac-Sia (green) (C) binding to IDV HEF (green, cartoon) contoured at 1.0 sigma, 1.0 sigma and 0.8 sigma, respectively, and the figures were drawn by Pymol software. The 2Fo-Fc maps were generated by FFT program in CCP4 software.
(TIF)

S2 Fig Sequence alignment of representative ICV and IDV HEFs: ICV HEF (C/Johannesburg/1/66), IDV HEF (D/swine/Oklahoma/1334/2011). HEF1 and HEF2 are shown in panel A and B respectively. The secondary structure elements are defined based on ESPript [73], and are labeled using our IDV HEF structure. The sequence logos were generated after the total 223 sequences of ICV HEF alignment or total 14 sequences of IDV HEF alignment to visualize the sequence conservation by Geneious [74]. All the sequences were obtained from the NIAID Influenza Research Database (IRD) online through the web site at http://www.fludb.org.
(TIF)

S3 Fig SDS-PAGE and western blot of the IDV HEF crystals, and the N-terminal amino acid sequencing result of the HEF2 band. (A) The SDS-PAGE of IDV HEF crystals shows that there are two bands, HEF1 and HEF2, confirming the IDV HEF protein had undergone proteolytic processing in the crystal form. Then western blot shows the HEF2 band using anti-his antibody. (B-F) Mass spectrometry maps of the first five N-terminal amino acids of the HEF2 band. The maps show the first five amino acids are IFGID. (G) Standard mass spectrometry map of different amino acids.
(TIF)

S4 Fig Electron density of the fusion peptide in the IDV HEF structure. The panels show portions of 2Fo-Fc electron density maps for the N terminal of HEF2 contoured at 1.0 sigma.
(TIF)

S1 Table Glycan microarray analysis of IDV HEF protein. (XLSX)

S2 Table Glycan microarray analysis of IDV and ICV HEF protein using a more extended glycan array. (XLSX)
