# Correction: An Open Receptor-Binding Cavity of Hemagglutinin-Esterase-Fusion Glycoprotein from Newly-Identified Influenza D Virus: Basis for Its Broad Cell Tropism

**DOI:** 10.1371/journal.ppat.1005505

**Published:** 2016-03-08

**Authors:** Hao Song, Jianxun Qi, Zahra Khedri, Sandra Diaz, Hai Yu, Xi Chen, Ajit Varki, Yi Shi, George F. Gao

The affiliation for the seventh author is incorrect. Ajit Varki is not affiliated with #4 but with #3 University of California, San Diego, CA, USA.

The following information is missing from the Funding section: This work was also supported by NIH grants R01GM32373 and U01 CA199792 (to AV).

There are errors in the Author Contributions. The correct contributions are: Conceived and designed the experiments: GFG YS HS JQ. Performed the experiments: HS JQ SD. Analyzed the data: HS JQ YS ZK. Contributed reagents/materials/analysis tools: HS JQ YS HY XC. Wrote the paper: HS AV YS GFG. Collected the X-ray data and solved the structures: JQ. Array analysis and interpretation: ZK SD AV. Synthesis of glycans for array: HY XC.
