# Supplementary figures and images for: An Open Receptor-Binding Cavity of Hemagglutinin-Esterase-Fusion Glycoprotein from Newly-Identified Influenza D Virus: Basis for Its Broad Cell Tropism
Source: PLoS Pathog. 2016 Jan 27;12(1):e1005411. doi: 10.1371/journal.ppat.1005411 (PMC4729479; doi:10.1371/journal.ppat.1005411)

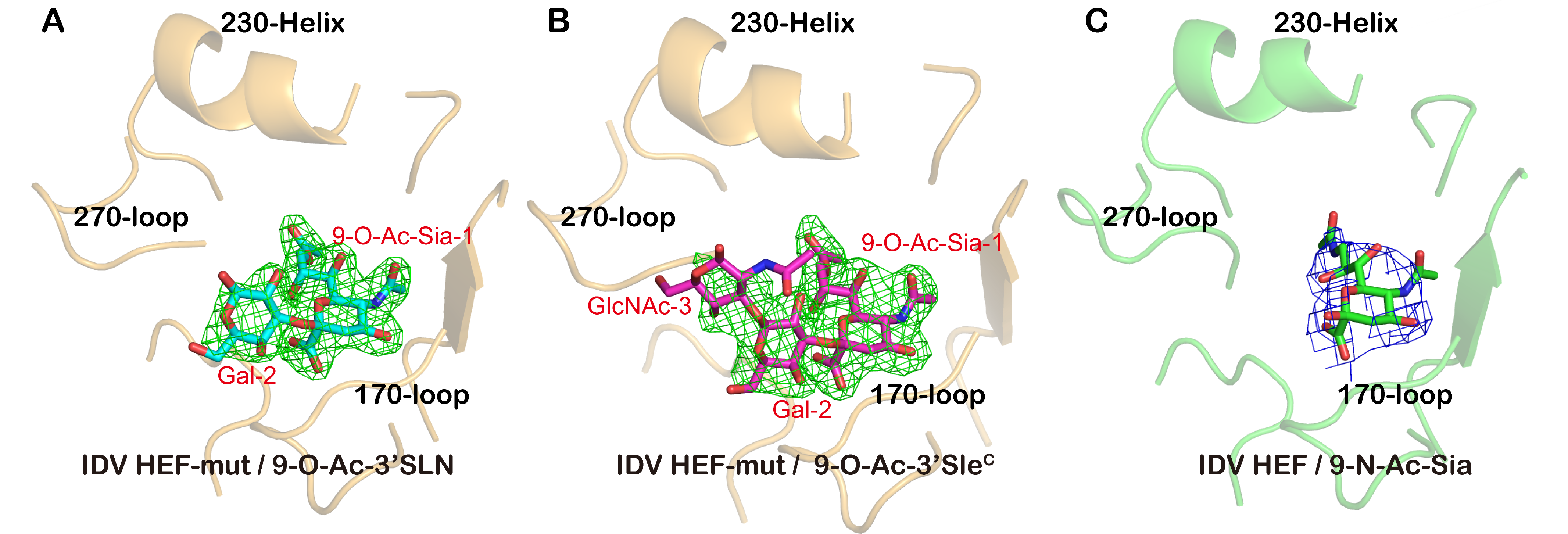

Supplement: S1 Fig — The panels show portions of 2Fo-Fc electron density maps for these glycan receptor analogs 9-O-Ac-3'SLN (cyan) (A) or 9-O-A-c3'SleC (magenta) (B) binding to IDV HEF-mut protein (orange, cartoon) and 9-N-Ac-Sia (green) (C) binding to IDV HEF (green, cartoon) contoured at 1.0 sigma, 1.0 sigma and 0.8 sigma, respectively, and the figures were drawn by Pymol software. The 2Fo-Fc maps were generated by FFT program in CCP4 software. (TIF) [file ppat.1005411.s001.tif]

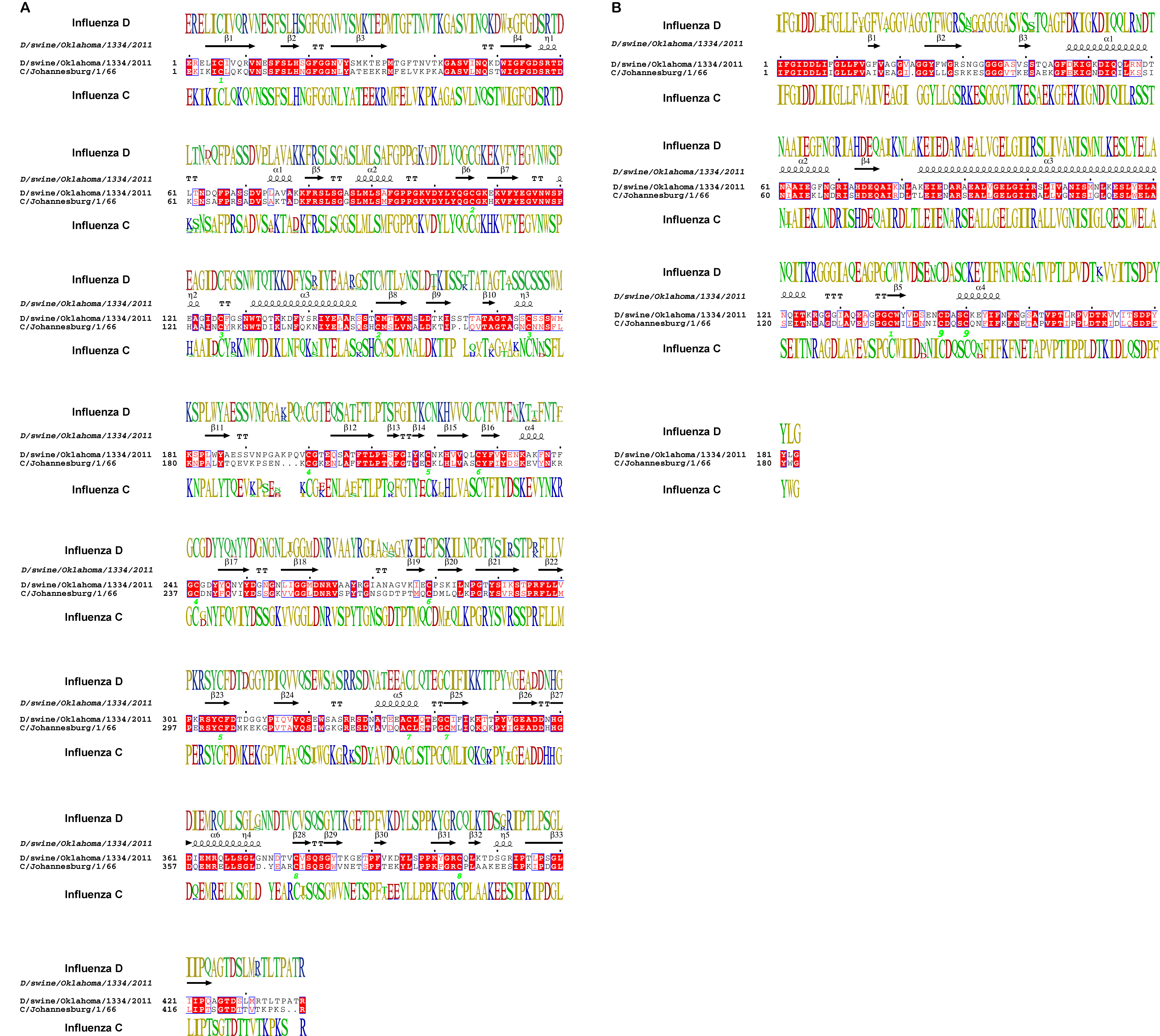

Supplement: S2 Fig — HEF1 and HEF2 are shown in panel A and B respectively. The secondary structure elements are defined based on ESPript [73], and are labeled using our IDV HEF structure. The sequence logos were generated after the total 223 sequences of ICV HEF alignment or total 14 sequences of IDV HEF alignment to visualize the sequence conservation by Geneious [74]. All the sequences were obtained from the NIAID Influenza Research Database (IRD) online through the web site at http://www.fludb.org. (TIF) [file ppat.1005411.s002.tif]

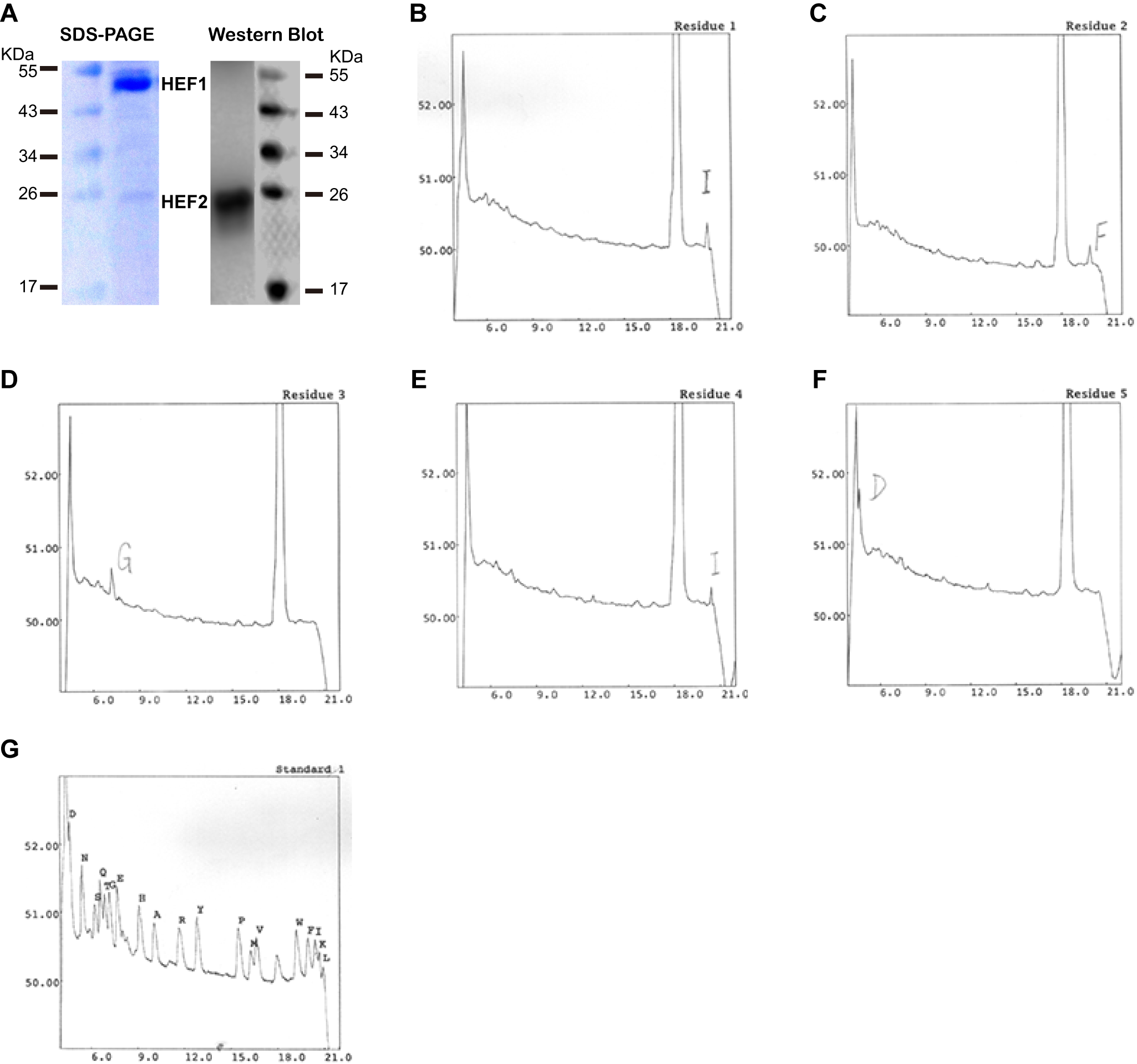

Supplement: S3 Fig — (A) The SDS-PAGE of IDV HEF crystals shows that there are two bands, HEF1 and HEF2, confirming the IDV HEF protein had undergone proteolytic processing in the crystal form. Then western blot shows the HEF2 band using anti-his antibody. (B-F) Mass spectrometry maps of the first five N-terminal amino acids of the HEF2 band. The maps show the first five amino acids are IFGID. (G) Standard mass spectrometry map of different amino acids. (TIF) [file ppat.1005411.s003.tif]

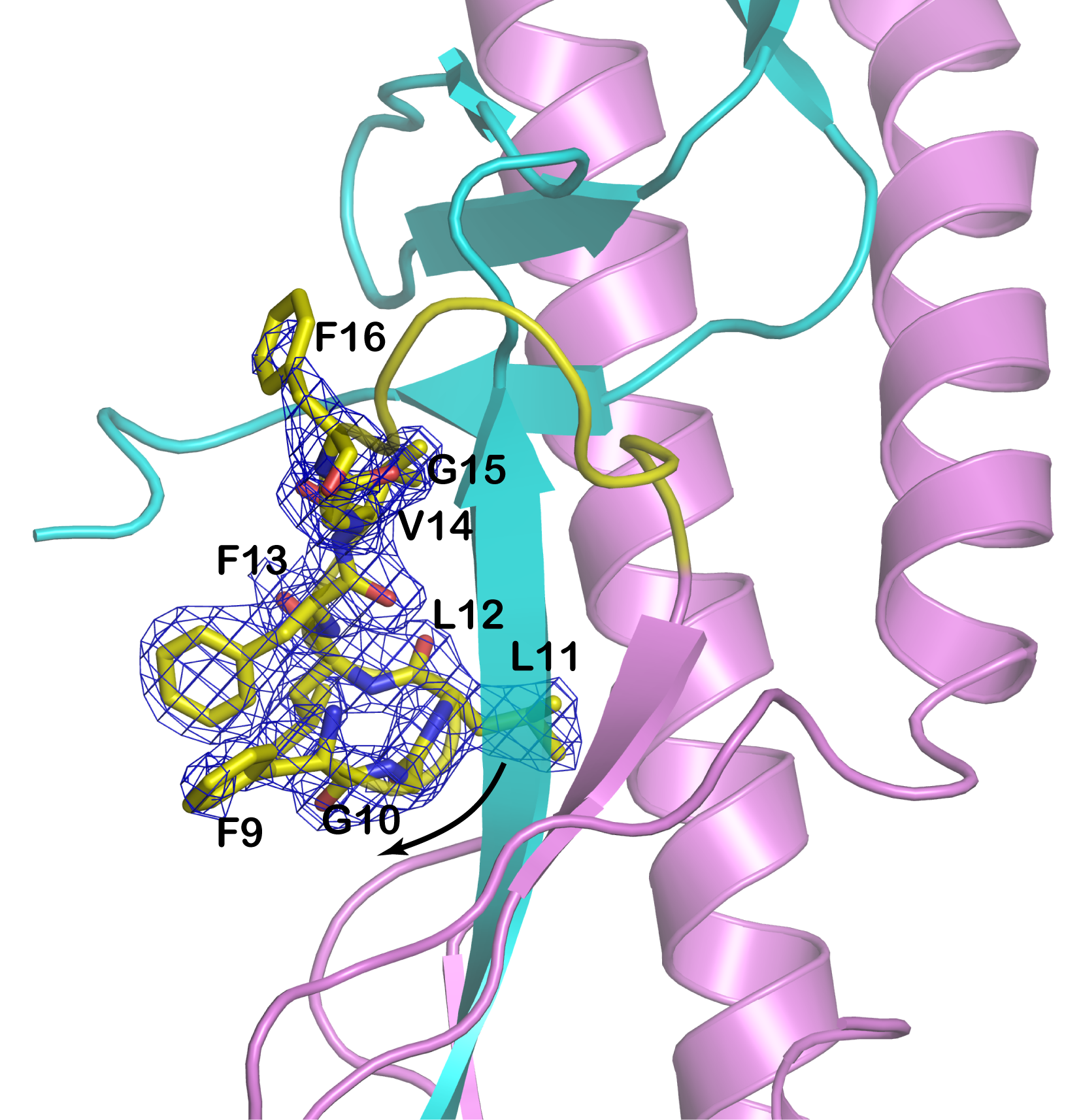

Supplement: S4 Fig — The panels show portions of 2Fo-Fc electron density maps for the N terminal of HEF2 contoured at 1.0 sigma. (TIF) [file ppat.1005411.s004.tif]
